# A Decade of Invasive Meningococcal Disease Surveillance in Poland

**DOI:** 10.1371/journal.pone.0071943

**Published:** 2013-08-20

**Authors:** Anna Skoczyńska, Izabela Waśko, Alicja Kuch, Marcin Kadłubowski, Agnieszka Gołębiewska, Małgorzata Foryś, Marlena Markowska, Patrycja Ronkiewicz, Katarzyna Wasiak, Aleksandra Kozińska, Bożena Matynia, Waleria Hryniewicz, and participants of of a laboratory-based surveillance of community acquired invasive bacterial infections (BINet)

**Affiliations:** National Reference Centre for Bacterial Meningitis, Department of Epidemiology and Clinical Microbiology, National Medicines Institute, Warsaw, Poland; University of Cambridge, United Kingdom

## Abstract

**Background:**

*Neisseria meningitidis* is a leading etiologic agent of severe invasive disease. The objective of the study was to characterise invasive meningococcal disease (IMD) epidemiology in Poland during the last decade, based on laboratory confirmed cases.

**Methods:**

The study encompassed all invasive meningococci collected between 2002 and 2011 in the National Reference Centre for Bacterial Meningitis. The isolates were re-identified and characterised by susceptibility testing, MLST analysis, *por*A and *fet*A sequencing. A PCR technique was used for meningococcal identification directly from clinical materials.

**Results:**

In the period studied, 1936 cases of IMD were confirmed, including 75.6% identified by culture. Seven IMD outbreaks, affecting mostly adolescents, were reported; all were caused by serogroup C meningococci of ST-11. The highest incidence was observed among children under one year of age (15.71/100,000 in 2011). The general case fatality rate in the years 2010–2011 was 10.0%. Meningococci of serogroup B, C, Y and W-135 were responsible for 48.8%, 36.6%, 1.2% and 1.2% of cases, respectively. All isolates were susceptible to third generation cephalosporins, chloramphenicol, ciprofloxacin, and 84.2% were susceptible to penicillin. MLST analysis (2009–2011) revealed that among serogroup B isolates the most represented were clonal complexes (CC) ST-32CC, ST-18CC, ST-41/44CC, ST-213CC and ST-269CC, and among serogroup C: ST-103CC, ST-41/44CC and ST-11CC.

**Conclusions:**

The detection of IMD in Poland has changed over time, but observed increase in the incidence of the disease was mostly attributed to changes in the surveillance system including an expanded case definition and inclusion of data from non-culture diagnostics.

## Introduction

*Neisseria meningitidis* is a leading etiologic agent of severe invasive disease characterized by rapid onset, of which meningitis and septicaemia are the most common and important manifestations [1], [2]. Meningococcal disease has a general case-fatality rate around 10%, however it is lower for meningitis (approximately 5%), and higher for septicaemia (from 5 to 40%, but even up to 70% in some studies) [1], [3]–[6]. Additionally, 11–19% of survivors may develop long-term sequelae [7], [8]. Although meningococcal infections may appear in every age group, infants and young children are at the highest risk of invasive meningococcal disease (IMD). A second peak of IMD incidence is observed in adolescence [9]. The disease may occur sporadically, as outbreaks or large epidemics; this last is characteristic for meningococci of serogroup A in Africa, and there are periodic fluctuations in IMD incidence and the occurrence of outbreaks and epidemics [1], [8]. In addition to the continuously changing epidemiology, IMD incidence and serogroup/clonal complex distribution are highly regional. Therefore, there is a necessity for comprehensive IMD monitoring to assess the local epidemiology and disease burden which may influence vaccine choice and prevention strategies, especially in light of serogroup- or protein-specific prophylaxis available [10].

The objective of the study was to characterise invasive meningococcal disease epidemiology in Poland during the last decade (2002–2011), based on laboratory confirmed cases and in particular to assess the IMD incidence, serogroup distribution, antimicrobial resistance patterns and molecular characteristics of isolates. This is the first such wide scale study on meningococcal isolates responsible for invasive infections in Poland.

## Materials and Methods

### Ethical Statement

Isolates were obtained as part of routine activity of the National Reference Centre for Bacterial Meningitis (NRCBM) and were analyzed anonymously. All data were collected in accordance with the European Parliament and Council decision for the epidemiological surveillance and control of communicable disease in the European Community [11], [12]. Ethical approval and informed consent were thus not required.

### Country Background

In Poland, IMD is a notifiable disease. Every suspected case has to be reported by physicians to the local Sanitary Inspectorate within 24 hours of hospital admission. IMD cases are registered through two independent surveillance systems, a written documentation-based surveillance system run by the National Institute of Public Health-National Institute of Hygiene (NIPH-NIH) and a laboratory-confirmed surveillance system run by the NRCBM. The registration system of meningococcal disease has changed over the study period (2002–2011). Until 2005, both Polish surveillance systems collected data on meningococcal meningitis only, although the NRCBM was receiving sporadically isolates responsible for other invasive infections. Since 2005, a mandatory notification of all IMD cases has been introduced [5]. Additionally, the NRCBM started using a routine non-culture PCR method for IMD laboratory identification from 2005 onwards. Finally, the NRCBM system was reinforced by building a voluntary-based network (BINet) of hospital laboratories more deeply engaged in the surveillance of community-acquired invasive bacterial infections including IMD, in Poland in 2008. For laboratories involved in BINet, both the shipment and diagnostics of isolates or clinical materials are offered free of charge. The NRCBM receives meningococcal isolates, as well as data on demographic characteristics, antibiotic therapy, vaccination status, clinical diagnosis, and disease outcome if already available. In the majority of cases the outcome is unknown at time of isolate/material shipment. Therefore, from 2010 the NRCBM has started to actively obtain information concerning outcome by phone calls to reffering microbiologists or physicians.

At the beginning of the study the total Polish population was 38,242,197, of whom 1,919,827 (5.0%) were children younger than 5 years of age (data for 31^st^ December 2001), while at the end of 2011, the population was 38,538,447, including 2,072,768 (5.4%) children under five years of age [http://demografia.stat.gov.pl/bazademografia/CustomSelect.aspx]. Estimates of the national census for 31^st^ December of every year were used as the denominator for the calculation of annual incidence rates [http://www.stat.gov.pl/gus/5840_655_PLK_HTML.htm?action=show_archive].

### Case Definition

A case of IMD was defined as the recovery of an isolate of *N. meningitidis* or meningococcal DNA from a normally sterile body site, such as blood, cerebrospinal fluid (CSF), joint aspirates, or tissues samples. In addition, isolates from the nose or nasopharynx were included in the analysis only if both, clinical symptoms of IMD were observed and positive PCR results were obtained from sterile body sites material. Only one isolate from each IMD case was included in the analysis.

### Identification and Serogrouping

All isolates were identified based on typical morphology of colonies, Gram stain, oxidase test and API NH test (bioMerieux, Marcy l’Etoile, France) or Rapid NH System (Remel) according to the manufacturer’s instructions. Serogroups A, B, C, W-135 and Y were determined by slide agglutination tests using commercial antisera (Remel).

The NRCBM has also been receiving clinical materials (blood, cerebrospinal fluid or tissues samples) from patients with suspected IMD. The DNA isolated from these samples was used for polymerase chain reactions to identify *N. meningitidis* to the species and capsular group level [13], [14].

### Susceptibility Testing

Minimal inhibitory concentrations (MICs) for penicillin, cefotaxime, chloramphenicol, rifampicin, and ciprofloxacin were determined by the broth microdilution method according to the Clinical and Laboratory Standards Institute (CLSI) guidelines up to June 2007, and subsequently by the E-test (AB Biodisk, Solna, Sweden) or MICEvaluators (Oxoid) methods according to manufacturers’ instructions [15] and interpreted according to the latest EUCAST guidelines [16]. *Streptococcus pneumoniae* ATCC 49619 strain was used as the quality control strain.

### Multilocus Sequence Typing and Sequencing of *por*A and *fet*A Genes

The meningococci were also characterised by multilocus sequence typing and DNA sequencing of the *porA* and *fetA* genes, which encode outer membrane proteins [17], [18]. Sequence types (ST) and PorA and FetA types were determined via the meningococcal typing website [http://pubmlst.org/neisseria/].

Chi square test or Fisher’s exact test were used to analyse the differences in frequencies (Programme STATISTICA 10 trial); p-values ≤0.05 were considered to be significant.

## Results

### Descriptive Epidemiology of Invasive Meningococcal Disease Cases

**Figure 1:**
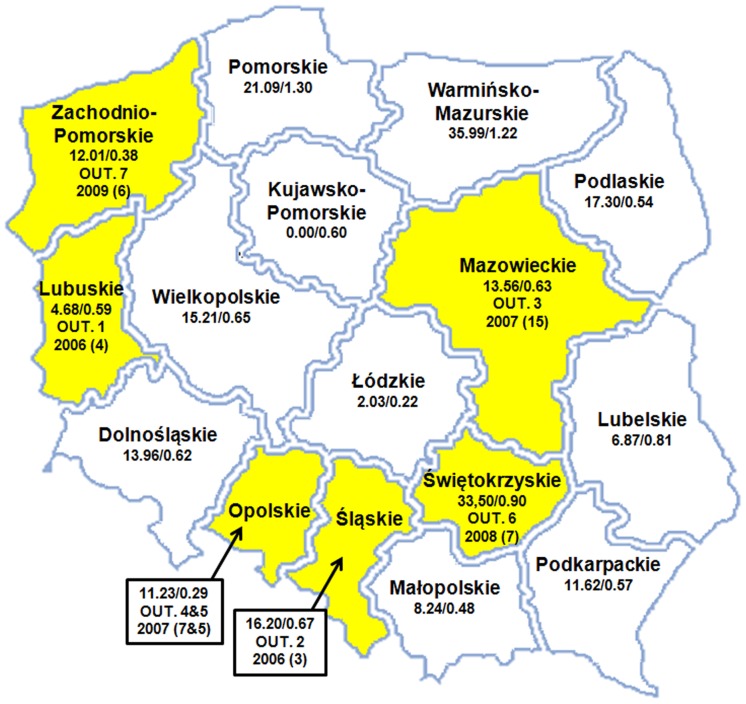
Map of Poland with the incidence rates (per 100,000) of invasive meningococcal disease in children under 1 year of age/all age groups, in 16 voivodeships, in years 2010–2011. The voivodeships with notified outbreaks during study period are marked in yellow (OUT. – outbreak, numbered like in the text, the year of the outbreak, in bracket there is number of outbreak cases).

Between 2002 and 2011, the NRCBM confirmed 1936 cases of IMD, including 1463 (75.6%) identified by culture and 473 (24.4%) by PCR. The number of laboratory confirmed cases per year increased over the study period from 35 in 2002 to 337 in 2007, but subsequently decreased to 293 in 2011. Data on age were available for 1903 cases (98.3%). The incidence rates of IMD by age group in specific years are shown in Table 1. The highest incidence was observed among children under one year of age (15.71/100,000 in 2011), amongst whom 21.2% of all IMD cases occurred, and was also high in children under 5 years of age (in total 7.04/100,000 in 2011). There was also a second incidence peak in patients aged 15–19 years old (Table 1). Meningococci were isolated in hospitals located in all 16 voivodeships (regions) of Poland, but isolate submissions to the NRCBM and consequently incidence rates of IMD differed significantly among voivodeships as is shown for years 2010–2011 in Figure 1.

**Table 1:** The total annual incidence rates (/100,000) of invasive meningococcal disease (IMD)/meningitis rates in age groups in Poland based on laboratory-confirmed cases, including culture- and PCR-positive samples, 2002–2011 (only cases with known age were included when calculating the incidence) and total annual incidence rates of IMD registered by the compulsory notification system (NIPH-NIH).

Age	2002	2003	2004	2005	2006	2007	2008	2009	2010	2011	%Cases^a^
**in months**											
**0–11**	3.97/3.40	3.15/1.72	7.91/5.08	5.24/3.86	8.32/4.56	15.02/9.32	16.94/11.86	15.59/8.40	11.14/7.02	15.71/9.27	21.2
**12–23**	0.83/0.83	2.27/0.85	4.30/2.58	2.54/1.69	5.24/3.31	6.98/3.22	10.09/6.73	6.28/4.59	7.17/4.78	8.21/4.83	11.0
**24–35**	0.53/0.53	1.38/1.10	1.14/0.85	3.44/2.29	2.26/1.70	3.31/1.38	3.76/1.88	6.72/3.36	3.62/1.93	4.84/3.00	6.3
**36–47**	0	0.53/0.53	1.10/0.55	1.42/1.42	1.72/1.72	5.37/2.54	3.04/1.38	5.10/3.49	3.62/2.58	4.17/3.24	5.1
**48–59**	0.26/0.26	0.26/0.26	0.80/0.53	1.93/1.66	0.85/0.57	2.58/0.86	2.55/1.41	2.21/1.66	2.15/1.07	2.97/1.98	3.2
**in years**											
**0–4**	**1.07/0.97**	**1.48/0.88**	**3.01/1.89**	**2.92/2.19**	**3.74/2.40**	**6.80/3.56**	**7.57/4.87**	**7.37/4.40**	**5.64/3.54**	**7.04/4.39**	46.8
**5–9**	0.18/0.18	0.09/0.05	0.39/0.24	0.40/0.20	0.89/0.57	1.40/0.91	1.65/0.94	1.12/0.78	0.90/0.51	0.88/0.60	7.7
**10–14**	0.11/0.07	0.30/0.23	0.28/0.16	0.37/0.25	0.35/0.13	1.26/0.86	0.85/0.47	1.03/0.93	0.35/0.30	0.78/0.57	6.5
**15–19**	0.12/0.09	0.13/0.13	0.70/0.27	0.31/0.28	0.97/0.79	1.81/1.26	1.91/1.22	1.46/1.07	0.79/0.54	1.90/1.60	13.9
**20–24**	0.03/0.03	0.03/0.03	0.09/0.06	0.24/0.15	0.43/0.33	1.13/0.66	0.75/0.52	0.64/0.57	0.46/0.28	0.61/0.50	7.1
**25–44**	0.02/0.02	0.03/0.02	0.06/0.02	0.05/0.03	0.09/0.07	0.29/0.16	0.29/0.22	0.24/0.19	0.17/0.14	0.20/0.15	8.4
**45–64**	0	0.03/0.02	0.04/0.03	0.07/0.05	0.10/0.08	0.33/0.22	0.21/0.14	0.16/0.11	0.14/0.13	0.21/0.17	7.0
**65+**	0.02/0.02	0.06/0.02	0.06/0.02	0.02/0.02	0.02/0	0.16/0.04	0.17/0.12	0.17/0.10	0.10/0.06	0.19/0.11	2.6
**All ages**	**0.09/0.08**	**0.13/0.09**	**0.28/0.15**	**0.26/0.19**	**0.40/0.28**	**0.88/0.52**	**0.86/0.56**	**0.77/0.53**	**0.54/0.37**	**0.76/0.53**	100
**No cases** b	35	51	106	99	154	337	327	294	207	293	1903
**% PCR+** c	0	0	0	7.1	11.7	27.6	34.3	34.4	23.7	27.6	24.2
**Men-cult.** d	0.08	0.09	0.15	0.17	0.25	0.39	0.37	0.33	0.28	0.38	0.08
**NIPH-NIH** e	**0.22**	**0.17**	**0.29**	**0.55**	**0.61**	**1.04**	**0.97**	**0.81**	**0.60**	**0.77**	

a %Cases **-** percentage of IMD cases in age group among all cases;

b No cases - total number of cases in a particular year;

c percentage of cases identified by PCR in a particular year;

d Men-cult. - incidence rates of meningitis confirmed by culture only;

e NIPH-NIH – total incidence of invasive meningococcal disease for all age groups registered by compulsory notification system run by the National Institute of Public Health – National Institute of Hygiene (Available: http://www.pzh.gov.pl/oldpage/epimeld/index_p.html. Accessed 2013 May 24).

Of 1936 *N. meningitidis* isolates, 1002 (51.8%) were recovered from CSF, 901 (46.5%) from blood, 25 (1.3%) from *post mortem* tissue samples, 7 (0.4%) from the nasopharynx, and 1 from joint fluid. Among all the patients, 42.4% were diagnosed as having meningitis, 22.3% with sepsis, 23.9% with meningitis and sepsis, and the remaining 11.5% with other manifestations or undetermined IMD. The patients’ age ranged from 1 day to 87 years (median, 6 years). Among 1920 patients with reported gender, 55.7% were male.

The case fatality rate (CFR) was assessed for IMD between 2010 and 2011, as the outcome was known for 91.6% of cases during this period, whereas in the years from 2002 to 2009 such information was available for 25.1% of cases only. CFR data are shown in Table 2. The general CFR was 10.0% for cases with known outcome only, and was highest in patients aged ≥65 years (46.2%, p = 0.001), although the incidence of IMD was lowest in that age group. The highest CFR was found in patients with sepsis (22.4%), as compared to patients with meningitis and sepsis (7.0%, p = 0.0007) and to meningitis alone (3.1%, p<0.0001).

**Table 2:** Incidence of invasive meningococcal disease (/100,000) case fatality rate (CFR) and percentage of cases with sequelae in age groups in Poland, 2010–2011 (only cases with known outcome were included when calculating the CFR, n = 458).

Age	Incidence	Total CFR (%)	MenBCFR^a^	MenCCFR^b^	Sequelae (%)
**in months**					
**0–11**	13.36	10.9	9.3	14.3	3.0
**12–23**	7.69	8.6	9.8	5.9	3.4
**24–35**	4.24	17.6	26.7	11.1	0.0
**36–47**	3.91	6.9	7.1	6.7	3.4
**48–59**	2.57	5.0	10.0	0.0	5.0
**in years**					
**0–5**	6.35	10.3	11.0	9.0	2.9
**5–9**	0.89	12.5	9.5	10.0	3.1
**10–14**	0.56	5.6	12.5	0.0	5.6
**15–19**	1.33	3.7	3.7	6.5	1.9
**20–24**	0.53	15.4	23.5	0.0	3.8
**25–44**	0.18	7.9	0.0	9.5	2.6
**45–64**	0.17	2.9	0.0	6.7	5.7
**65+**	0.14	46.2	37.5	50.0	7.7
**All ages**	**0.65**	**10.0**	**10.3**	**8.8**	**3.3**

a MenB CFR - case fatality rate associated with infections caused by serogroup B meningococci;

b MenC CFR - case fatality rate associated with infections caused by serogroup C meningococci.

Establishment of BINet contributed to a slight increase of IMD detection by laboratories involved in the network, from 60.8% before 2008 to 61.8% since 2008 and increase of IMD detected by PCR, from 59.1% before 2008 to 63.4% since 2008. However, these values were not statistically significant.

### Serogroup Distribution among *N. meningitidis*

**Figure 2:**
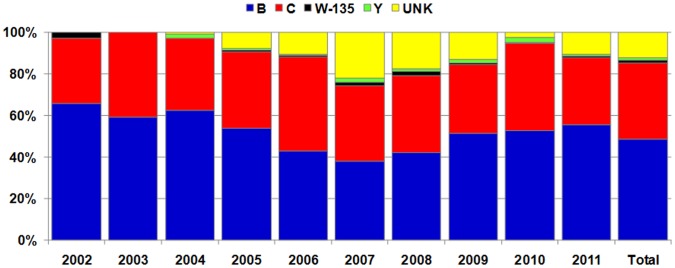
Serogroup distribution of meningococci responsible for invasive infections in Poland, 2002–2011. (n = 1936; UNK - cases with unknown serogroup).

The serogroup was identified for 1700 (87.8%) cases, including 1445 IMD cases confirmed by culture and 255 by PCR. Out of all meningococcal cases, 944 (48.8%) were caused by serogroup B (MenB), 709 (36.6%) by serogroup C (MenC), 24 (1.2%) by serogroup Y (MenY) and 23 (1.2%) by serogroup W-135 (MenW-135). The serogroup was not identified or causative isolates were nongroupable in 236 (12.2%) cases, of which 92.4% were identified by PCR. Serogroup distribution of meningococci responsible for invasive infections in Poland between 2002 and 2011 is presented in Figure 2. The data on the incidence of IMD caused by isolates of particular serogroups and serogroup distribution by age groups over the years 2002–2006 and 2007–2011 is shown in Table 3. The results were separated because the incidence rates were significantly different for the two periods.

**Table 3:** Incidence rates (/100,000) of invasive meningococcal disease caused by isolates of particular serogroups and serogroup distribution in age groups in Poland, 2002–2006 and 2007–2011.

	Incidence per100,000/serogroup distribution in age group, %									
	2002–2006					2007–2011				
Age	B	C	W-135	Y	UNK^a^	B	C	W-135	Y	UNK^a^
**in months**										
**0–11 m**	4.30/74.8	1.12/19.4	0.11/1.9	0/0	0.22/3.9	9.72/65.3	3.17/21.3	0.10/0.7	0.10/0.7	1.78/12.0
**12–23 m**	1.91/63.0	0.90/29.6	0/0.0	0.06/1.9	0.17/5.6	4.99/64.5	1.84/23.9	0.05/0.6	0/0	0.85/11.0
**24–35 m**	0.84/48.4	0.72/41.9	0.06/3.2	0/0	0.11/6.5	2.18/48.9	1.78/39.8	0.10/2.3	0/0	0.41/9.1
**36–47**	0.49/52.9	0.33/35.3	0/0	0.05/5.9	0.05/5.9	1.83/43.2	1.83/43.2	0/0	0.05/1.2	0.52/12.3
**48–59 m**	0.38/46.7	0.43/53.3	0/0	0/0	0/0	1.30/52.2	0.71/28.3	0/0	0/0	0.49/19.6
**in years**										
**0–4**	1.57/64.5	0.70/28.6	0.03/1.4	0.02/0.9	0.11/4.5	4.09/59.4	1.89/27.5	0.05/0.7	0.03/0.4	0.82/11.9
**5–9**	0.20/53.8	0.16/41.0	0/0	0/0	0.02/5.1	0.46/38.9	0.47/39.8	0/0	0/0	0.25/21.3
**10–14**	0.08/28.6	0.20/71.4	0/0	0/0	0/0	0.23/27.0	0.48/55.1	0.01/1.1	0.02/2.2	0.13/14.6
**15–19**	0.13/30.8	0.24/55.4	0/0	0.01/1.5	0.05/12.3	0.48/30.2	0.84/53.3	0.02/1.5	0.04/2.5	0.20/12.6
**20–24**	0.07/40.7	0.09/55.6	0/0	0/0	0.01/3.7	0.29/39.8	0.22/29.6	0.03/4.6	0.02/2.8	0.17/23.1
**25–44**	0.03/53.8	0.02/46.2	0/0	0.0	0/0	0.08/34.6	0.11/48.1	0.00/0.8	0/0	0.04/16.5
**45–64**	0.03/58.3	0.01/25.0	0/0	0.00/4.2	0.01/12.5	0.11/51.4	0.07/30.6	0.00/1.8	0.01/4.5	0.02/11.7
**65+**	0.02/55.6	0.02/44.4	0/0	0/0	0/0	0.09/57.5	0.05/30.0	0.00/2.5	0.01/5.0	0.01/5.0
**All ages**	**0.12/53.3**	**0.09/39.8**	**0.00/0.7**	**0.00/0.9**	**0.01/5.4**	**0.36/47.5**	**0.27/35.9**	**0.01/1.2**	**0.01/1.4**	**0.11/13.9**

a UNK – cases with unknown serogroup.

The case fatality rate associated with MenB infections was slightly higher (10.3%) than with MenC (8.8%, p = 0.73) but this finding was not statistically significant (Table 2).

### Antimicrobial Susceptibility

Antimicrobial susceptibility testing was performed for 1373 meningococcal isolates. Most of them (n = 1156, 84.2%) were highly susceptible to penicillin, however, 197 (14.3%) showed intermediate susceptibility, and 20 (1.5%) resistance. In general MenB were more often non-susceptible to penicillin (19.0%) than those of MenC (11.3%, p = 0.0002). Although not statistically significant, non-susceptibility to penicillin was the most common in children under one year of age, in whom MenB infections were the most frequent in comparison to the whole population (36.6% vs 28.9%, p = 0.24) All isolates tested were susceptible to third generation cephalosporins, chloramphenicol and ciprofloxacin. Only 4 isolates (0.3%) were resistant to rifampicin, including 2 of MenB with MICs of 0.5 and 0.75 mg/l, respectively, one of MenC with an MIC of 0.5 mg/l and one of MenW-135 with an MIC of >32 mg/l.

MIC, MIC_50_ and MIC_90_ ranges (in mg/l) were as follows: for penicillin < = 0.0075–1.0, 0.03–0.06 and 0.06–0.012, respectively; for third generation cephalosporins < = 0.0008–0.12, 0.0016–0.004 and 0.003–0075, respectively; for chloramphenicol, 0.25–2.0, 0.5–2 and 1–2, respectively: for ciprofloxacin < = 0.002–0.015, 0.002–0.015 and 0.004–0.015, respectively; for rifampicin 0.002->32, 0.008–0.06 and 0.047–0.25, respectively. Analysis of meningococcal susceptibility did not reveal any significant differences in their percentages, values of MIC_50_ and MIC_90_, and MICs ranges, through the whole period studied.

Based on data between 2010 and 2011, infections caused by isolates non-susceptible to penicillin were not associated with higher CFR in comparison to susceptible ones (9.8% vs 8.5%, p = 0.89).

### Molecular Characteristics

MLST analysis, *por*A and *fet*A sequencing started to be routinely performed in the NRCBM since 2009 and therefore molecular analysis included isolates collected between 2009 and 2011. MLST analysis revealed 164 STs among MenB isolates, of which 79.1% belonged to eighteen international clonal complexes (CC). Despite the diversity, five major CCs (ST-32CC, ST-18CC, ST-41/44CC, ST-213CC and ST-269CC) grouped 73.5% of all MenB isolates studied.

**Figure 3:**
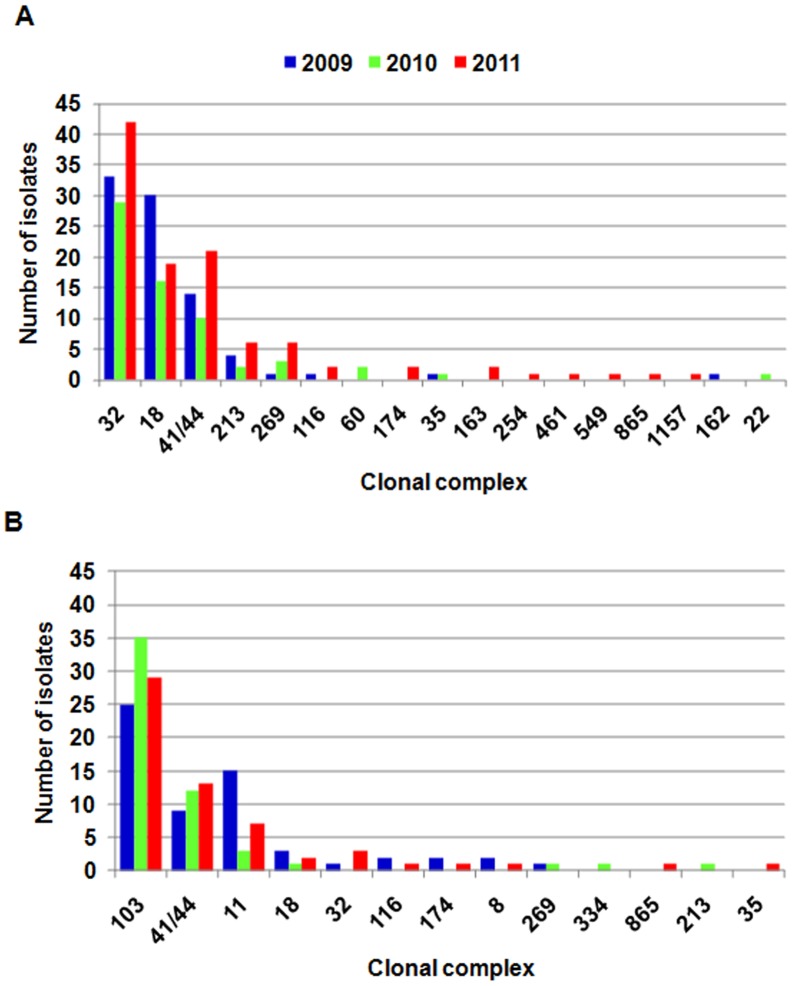
Distribution of clonal complexes between 2009 and 2011 among Polish meningococci of serogroup B (A) and C (B).

Among MenC isolates 58 STs were identified. Although 81.6% of MenC meningococci belonged to thirteen international clonal complexes (CC), 69.8% were grouped in three of the most predominant CCs (ST-103CC, ST-41/44CC and ST-11CC). Meningococci of ST-11CC and ST-103CC belonged exclusively to serogroup C and of ST-32CC, ST-18CC, ST-213CC and ST-269CC mostly to serogroup B, whereas ST-41/44CC meningococci belonged to both serogroups. Molecular characterization of the most represented clonal complexes (with at least 10 isolates) of MenB and MenC is summarized in Table 4. The distribution of CCs among Polish meningococci of MenB and MenC between 2009 and 2011 is shown in Figure 3.

**Table 4:** Molecular characterization of meningococcal isolates of serogroup B and C belonging to the most dominant clonal complexes (CC) represented at least by 10 isolates, 2009–2011.

	Serogroup B (n = 321)					Serogroup C (n = 212)		
Clonal complex (CC)	ST-32CC	ST-18CC	ST-41/44CC	ST-213CC	ST-269CC	ST-103CC	ST-41/44CC	ST-11CC
Number of isolates in CC	104	65	45	12	10	89	34	25
% B or C	32.4%	20.2%	14.0%	3.7%	3.1%	42.0%	16.0%	11.8%
ST^a^ (n)	15	42	19	4	9	4	14	3
Most common ST	ST-32 (71)	ST-145 (17)	ST-1194 (13)	ST-213 (7)	ST-479 (2)	ST-5133 (85)	ST-3346 (8). ST-5323 (8)	ST-11 (23)
PorA VR1,VR2 variants (n)	12	22	21	2	7	5	11	6
Most common PorA VR1,VR2								
combination (n)	7, 16 (57)	22, 14 (17)	7-2, 4 (12)	22, 14 (11)	19-1,15-11 (3)	18-1, 3 (65)	17, 16-4 (17)	5, 2 (14)
FetA variants (n)	8	25	11	3	7	2	6	2
Most common FetA variant (n)	F3-3 (85)	F1-17 (10)	F1-5 (29)	F5-5 (10)	F5-1 (3)	F3-9 (86)	F3-9 (18)	F3-3 (14)

a ST – sequence type.

Between 2009 and 2011, four isolates of MenW-135 were identified which belonged to four different STs. Only one of these belonged to a recognized international CC, ST-22CC. Sequencing of *por*A and *fet*A genes also confirmed the heterogeneity of the isolates. All five MenY belonged to international CCs: ST-22CC, ST-23CC, ST-92CC and ST-167CC (n = 2). Three MenY isolates shared the same PorA VR1 (5-1) and FetA (F4-1), and two the same VR2 (10-1).

Geographic analysis of clonal complex prevalence revealed that isolates of ST-11CC were more often responsible for infections in the West of Poland than in the East (7.1% vs 1.6%, p = 0.006), especially in Wielkopolskie and Zachodnio-Pomorskie voivodeships (in which the last documented outbreak took place) [19]. Representatives of this clone were also more often identified among isolates from 2009 in comparison to 2010 (8.1% vs 1.9%, p = 0.03).

Meningococci of ST-11CC were more frequently isolated from persons over 5 years of age (8.2%, p = 0.0004) and in particular from individuals 15–24 years old (13.7%, p<0.0001) in comparison to children under 5 years of age (1.1%). In contrast, meningococci of ST-18CC more often affected children under 5 years of age than persons over 5 years old (18.2% vs 7.8%, p = 0.003). This last CC was associated with the highest CFR (26.3%) in comparison with the whole Polish meningococcal population (8.7%, p = 0.002); however, this high CFR was not associated with any particular ST within ST-18CC. Although not statistically significant, the CFR was slightly higher for infections caused by isolates belonging to any of the recognised CCs (9.2%) compared to those not associated with any CCs (6.3%). Interestingly, non-susceptibility to penicillin was most prevalent among the isolates not belonging to any international CC (47.8% vs 28.8%, p = 0.003) and least prevalent among meningococci of ST-103CC, in comparison to the whole meningococcal population (4.9% vs 28.8%, p = 0.003).

For other CCs there were no significant differences in respect to CFR, geographical, temporal and age group distribution.

### IMD Outbreaks

Between 2002 and 2011, seven outbreaks were notified in Poland, affecting mostly adolescents [19]–[22]. All of them were caused by MenC of ST-11.

The ST-11 isolates have been notified in Poland since 1998 but until 2006 were responsible for 0–2 cases annually. In March 2006, the first outbreak (OUT.1 in the Figure 1) with 4 cases in army recruits took place at a military base in Lubuskie voivodeship in the West of Poland [22]. In June 2006 IMD was diagnosed in three teenage friends in Bytom (Śląskie voivodeship, OUT.2) based on characteristic clinical symptoms. The cases were not laboratory confirmed due to sampling being delayed until the commencement of antibacterial therapy, however ST-11 meningococci were isolated from the nasopharynx from four of their close contacts. Consequently, it was assumed that isolates of that clone were responsible for the outbreak [20]. In January 2007, 15 cases of IMD, including 2 fatal at a Warsaw military base (Mazowieckie voivodeship, OUT.3) were laboratory confirmed primarily using PCR or an antigen test. MLST analysis of five cases confirmed ST-11 meningococci [20]. In 2007, outbreaks of IMD were notified in two counties, Brzeg (7 cases, OUT.4) and Kluczbork (4 cases, OUT.5), in Opolskie voivodeship. Molecular analysis of available isolates revealed meningococci of ST-11 [21]. At the beginning of 2008, an outbreak (OUT.6) of 7 IMD cases, also caused by ST-11, was reported in Świętokrzyskie voivodeship. All of the above outbreaks were caused by ST-11 meningococci with a PorA variant P1.5-1,10-1, while those identified in Poland between 1997 and 2005 had PorA variant P1.5,2 [19].

Finally, in an outbreak (OUT.7) in Goleniów county of Zachodnio-Pomorskie voivodeship in March 2009, 6 IMD cases in individuals aged from 7 to 25 years of age were caused by ST-11. Interestingly, the Goleniów county isolates had an FetA variant (F3-3) that differed from all sequenced ST-11 isolates responsible for IMD cases before 2009 in Poland (generally, these were F3-6, with only two isolates having F3-9). Additionally, the isolates responsible for the Goleniów county outbreak had the same PorA variant as that of ST-11 meningococci isolated before 2006 (P1.5,2) [19]. Geographical sites of outbreaks are presented in Figure 1.

At two military bases and four other locations – two each in Opolskie, Świętokrzyskie and Zachodnio-Pomorskie voivodeships - vaccination campaigns against MenC were organized resulting in a high vaccine coverage [19]–[22].

## Discussion

The epidemiology of IMD is very dynamic and characterised by significant variation in the incidence, serogroup and sequence type/clonal complex distribution [23]. It is influenced by the patients’ age, geographic region, season, period studied and vaccination policy. Consequently, continuous laboratory-based surveillance of meningococcal isolates is needed in order to ensure appropriate IMD management and control. IMD laboratory-based surveillance in Poland has revealed a remarkable increase in IMD cases over the last decade. However, despite possible changes in the epidemiology, the rise in IMD has mainly been influenced by changes to the Polish surveillance system. Up to 2005, only meningococcal meningitis cases were notified in Poland; however, mandatory notification of all invasive cases has been required since then [5], [24]. Additionally, the NRCBM introduced a routine PCR-based diagnostic test (i.e. not culture-based) for identification of IMD in 2005. PCR is more sensitive and provides a more-accurate estimation of the incidence as shown by the fact that during the last five years 24–34% of all IMD cases in Poland were confirmed by this technique alone. Such increases in IMD detection following PCR introduction were also observed in other countries [10], [25], [26]. Finally, the laboratory-based system was reinforced by BINet activity in 2008 [27], [28]. The reinforcement due to the establishment of BINet did not result in a major increase in laboratory notifications of IMD. It could be explained by the fact that the medical community and general public were already very much alerted following six high-profile IMD outbreaks between 2006 and 2008, and the associated educational campaigns, that occurred immediately prior to the start of BINet activity [20]–[22]. All of the above mentioned formal changes in the Polish notification system resulted in an expected increase in overall IMD incidence rates. However, the simultaneous increase in culture-confirmed meningitis incidence rates may have been due to better surveillance and/or possible changes in IMD epidemiology in Poland (Table 1).

Between 2005 and 2011 Poland ranked among countries with an overall low IMD incidence rates ranging from 0.26 to 0.88 per 100,000 inhabitants. In 2009, IMD incidence rates in Europe varied widely between countries based on an ECDC assessment, ranging from 0.13 to 3.01 per 100,000 inhabitants, giving an average of 0.98 per 100,000 inhabitants [29]. As expected, the highest incidence of IMD was observed in children. The results of our study showed that 46.8% of all cases affected children under five years of age, including 21.2% of children aged less than one year. The general laboratory-confirmed incidence rate of IMD, 6.88/100,000 in Polish children under five years of age in the years from 2007 to 2011 was similar to that observed in Europe in 2009 (7.37/100,000) [29]. However, the average IMD incidence rate 14.87/100,000 between 2007 and 2011 in children aged under one year was lower in comparison to the average data for 27 European countries from 2006 (approximately 20/100,000) [10]. It is worthy emphasizing that geographic analysis revealed high variations in IMD incidence rates in children under one year of age between Polish regions (0.0 vs 35.99/100,000). This may be due to a diverse epidemiological situation, but more probably results from differences in the effectiveness of the monitoring system in place, which is strongly affected by insufficient/low frequency of blood culturing and blood samples submission to the NRCBM for PCR diagnostic [27], [30].

Universal vaccination against IMD has so far not been implemented in Poland, although the anti-meningococcal conjugated vaccines are registered and highly recommended. Until 2002, more than 85% of IMD cases in Poland were caused by MenB but from that year onwards a country-wide increase of IMD due to MenC was observed reflecting the rapidly changing epidemiology of this disease [31]. Our results also revealed that most cases of meningococcal disease are caused by MenB, although in some years of the study the proportions of serogroup B and C isolates were comparable. Similarly, MenB and MenC disease dominated in Europe at the end of the 20th Century; however, MenB are currently responsible for more than 85% of infections in countries with mass vaccination against MenC [32]–[34]. In our study, MenB infections predominated in children less than 1 year of age, but despite this the incidence of MenC disease was also highest in that age group in comparison to the other age groups. Such observations were also characteristic for other European countries [34], [35]. Unexpectedly, a rapid rise in the number of MenY infections was noticed in some countries, in both recent and previous studies, although our results did not demonstrate such an increase in Poland [36]–[38].

Even with limited data on disease outcome, our study showed that fatality differs by patient age and the manifestation of infection, but is independent of non-susceptibility to antibiotics as previously described [39]. The overall CFR found during our study (10.0%) was higher than the mean CFR of 7.8% found in other European countries. The CFR associated with MenB infections was higher (10.3% vs 6.5%), whereas that related to MenC was lower (8.8% vs 12.3%) in our study in comparison to the European average [35]. Surprisingly, in our study a low percentage of sequelae was notified in comparison to other authors, which is difficult to explain but it may be associated with poor reporting of sequelae or with reporting of very severe sequelae only [7], [8].

Most of the Polish meningococcal isolates were highly susceptible to penicillin; however, 15.8% of them showed some degree of decreased susceptibility to this antibiotic and the proportion of these strains has been gradually increasing [40]. The proportion of such isolates varies widely from country to country [41]. During our study we found four isolates that were resistant to rifampicin according to the latest EUCAST criteria [16]. However, according the latest CLSI criteria, as well as criteria proposed by the authors of a multi-centre study to establish the breakpoint for rifampicin resistance in meningococci by *rpoB* sequencing, rifampicin susceptible isolates should be defined as those with MICs of <1 mg/l. Using this breakpoint, only one of the isolates had an MIC of >32 mg/l, which would make it resistant to rifampicin [15], [42].

As shown in other countries, the population of MenB analysed during our study was much more diverse than the population of MenC meningococci [10]. Despite that, the majority of Polish MenB belonged to well known and widely distributed clonal complexes, namely ST-32CC, ST-18CC and ST-41/44CC [33,34; http://pubmlst.org/neisseria/]. Meningococci of the ST-32 complex, have been responsible for many epidemic and sporadic IMD cases worldwide, for example in Norway, Spain, Cuba, Brazil, Oregon in the USA and Normandy in France [43]–[45]. Isolates of ST-32 with PorA VR1,VR2 combination 7, 16 and FetA F3-3 of our study were similar to an epidemic clone found in Norway and Oregon (B:15:P1.7,16), which was confirmed by serotyping some of the Polish ST-32 MenB isolates using an ELISA (data not shown).

ST-18CC was very diverse given that 42 STs, 22 PorA VR1,VR2 combinations and 25 FetA variants were found amongst 65 isolates. Meningococci of this clonal complex were responsible for more than 3 times higher CFR in comparison to the whole Polish meningococcal population.

As previously mentioned, an increase in the proportion of MenC among all IMD cases has been observed since 2002. This was related to the rise of cases caused by meningococci of two clonal complexes ST-8CC and ST-103CC, which were not previously identified in Poland, apart from one case due to ST-8CC meningococci in 2001. Interestingly, the emergence of ST-8CC in some countries resulted in mass immunization campaigns, but also in a significant increase in the level of penicillin non-susceptibility [46]–[48]. ST-8CC isolates, which predominated among Polish MenC between 2003 and 2004, were almost universally susceptible to penicillin and in later years were responsible for no more than one or two cases annually ([49], unpublished NRCBM data). Such a decline, following its previous predominance, was also noted in Italy [50]. On the other hand, isolates of ST-103CC were the most common amongst MenC in Poland in 2005 and then from 2008 onwards (unpublished NRCBM data). In our study these isolates constituted 42% of all MenC. To begin with, the ST-103CC group was very homogenous and was composed exclusively of ST-5133 isolates with the same PorA and FetA profile. However, this group has diversified over time, with single and double locus variants of ST-5133 isolates not belonging to ST-103CC subsequently occurring. Interestingly, representatives of this clonal complex, although of different sequence types, were often responsible for infections and occasionally for IMD outbreaks in Brazil [51], [52].

In our study, the third most common clonal complex of MenC was the hyper-virulent ST-11 CC, which between 2006 and 2007 predominated among MenC in Poland and additionally in the years from 2006 to 2009 were responsible for numerous outbreaks. Infections with ST-11 meningococci, as also described by others, most commonly present as sepsis, affect mostly adolescents and young adults, are generally associated with a high CFR and have been responsible for numerous epidemics and outbreaks in different parts of the world [39], [53]–[57]. Similar to ST-103CC, ST-11CC in this study was also very homogenous by MLST analysis and was composed mainly of ST-11 isolates. However, during the study period, both PorA and FetA variants of ST-11 isolates appeared, which may partially explain the increase in sporadic cases as well as the occurrence of outbreaks in Poland. It has been suggested that even very small alterations in antigenic characteristics, which can be the result of a single point mutation, may result in an increase in the number of IMD cases in particular geographic areas [19], [55], [58].

The results of our study showed that the detection of IMD in Poland has changed over time, but observed increase in the incidence of the disease was mostly attributed to changes in the surveillance system, including an expanded case definition and the inclusion of data from non-culture diagnostics. Even though IMD is a rare disease in our country, the severity of the disease itself and the occurrence of outbreaks have resulted in a certain fear among the general public. To control IMD in Poland, where mass vaccination against the disease has yet to be introduced, inclusion of vaccines against MenC and MenB into the childhood immunization schedule should be considered.
